# Evaluating the Efficacy of Capacitive Resistive Monopolar Radiofrequency Combined With Proprioceptive Neuromuscular Facilitation in Managing Chronic Low Back Pain: A Randomised Controlled Trial

**DOI:** 10.1002/pri.70009

**Published:** 2024-11-21

**Authors:** Ivan Jurak, Silvija Grabar, Nikolino Žura, Lukrecija Jakuš

**Affiliations:** ^1^ Department of Physiotherapy University of Applied Health Sciences Zagreb Croatia; ^2^ Department of Physiotherapy Polyclinic Cerebellum Varaždin Croatia; ^3^ Department of Rheumatology and Rehabilitation KBC Zagreb Zagreb Croatia

**Keywords:** combined modality therapy, low back pain, pain management, quality of life

## Abstract

**Background and Purpose:**

This randomised controlled trial evaluates the effectiveness of capacitive resistive monopolar radiofrequency (CRMRF) combined with proprioceptive neuromuscular facilitation (PNF) training in managing chronic low back pain (CLBP). Given the multifactorial nature of CLBP, this study explores a multimodal treatment approach integrating CRMRF, known for its thermal effects and ability to alleviate pain through improved cell metabolism and microcirculation, with PNF training, which enhances muscle strength, flexibility, and proprioception.

**Methods:**

This study was designed as a single‐blind, parallel, randomised controlled trial conducted in an outpatient clinical setting. Over the course of four months, 62 participants, suffering from chronic low back pain were randomly assigned to receive either the combined CRMRF and PNF treatment or PNF alone, with primary outcomes measured in terms of pain and functional disability using the Visual Analogue Scale (VAS), Oswestry Disability Index (ODI). For secondary outcome of disability associated with pain, Quebec Pain Disability Scale (QPDS) and Roland‐Morris Disability Questionnaire (RMDQ) were used. The study's hypothesis was that the combined treatment would reduce pain and disability more effectively than PNF alone.

**Results:**

Results indicated that the experimental group experienced greater improvements in pain and functional disability, surpassing the minimally clinically important difference (MCID) for the VAS, ODI, QPDS and RMDQ, suggesting the clinical relevance of the combined CRMRF and PNF approach.

**Discussion:**

These findings are consistent with previous research highlighting the benefits of CRMRF in various musculoskeletal disorders and suggest that integrating CRMRF with PNF training offers a promising non‐invasive treatment option for CLBP sufferers. Overall, our study contributes to the growing evidence base supporting innovative, multimodal treatment strategies for managing CLBP, with the potential to enhance patients' quality of life.

**Trial Registration:** ClinicalTrials.gov identifier: NCT05682287

## 1 Introduction

Globally, low back pain (LBP) is a widespread musculoskeletal condition and the global burden of disability associated to this condition has been increasing particularly within the working‐age population (Wu et al. 2020). It has been reported that between 2% and 48% of patients with acute LBP in primary care settings transition to chronic LBP; these data are in line with a reported overall 32% transition rate to chronic LBP at 6 months (Stevans et al. 2021). Chronic low back pain (CLBP) is defined as pain, muscle tension, or stiffness lasting over 12 weeks, or as recurrent episodes occurring twice a year, each lasting over 24 h with at least 30 days pain‐free interval between them (Stanton et al. 2010). Given the multifactorial nature of CLBP, a supported multimodal treatment approach combines various analgesic agents with exercise therapy (ET)—as a first‐line treatment—and other physical modalities (Müller‐Schwefe et al. 2017).

A recent review found low to moderate quality evidence supporting the efficacy of proprioceptive neuromuscular facilitation (PNF) training in managing pain and disability in LBP patients, suggesting PNF as a useful strategy for chronic LBP management. PNF training has been recommended for sensorimotor control training as well as for stimulating lumbar muscle proprioception (Arcanjo et al. 2022). Basic procedures, such as rotational patterns of movement and different techniques, including rhythmic stabilisation, dynamic reversals, combination of isotonics, repeated contractions, and contract‐relax, can be applied to improve flexibility, muscle strength, and movement. Thus, the concept of PNF is to enhance joint coordination, muscle strength, movement control, stability, and mobility (Smedes et al. 2016). The most common types of PNF techniques used in the literature for LBP patients consist of rhythmic stabilisation and a combination of isotonics (Pourahmadi, Sahebalam, and Bagheri 2020).

Electrophysical agents based on radiofrequency are widely used in clinical practice due to their thermal effects, relieving pain and inflammation. The use of radiofrequency can be successful in reducing several chronic pain conditions such as chronic low back pain (Racz and Ruiz‐Lopez 2006; Van Boxem et al. 2008). The triple mechanism of action of radiofrequency is described: the sub‐thermal effect induces movement in the extracellular matrix, facilitating cell nutrition and improving cell metabolism; improved microcirculation and vasodilatation, favours tissue drainage and improved cell oxygenation and increased cell metabolism results in better and faster regeneration. Tissue repair creates quality tissue, which prevents the injury recurring (Racz and Ruiz‐Lopez 2006). The analgesic effect of radiofrequency can be explained according to Melzack's and Wall's Gateway Theory that Aβ nerves activated by thermal stimulation of capacitive resistive radiofrequency currents reduce the transmission of painful information and, consequently, pain intolerance is reduced. Also, vasodilatation induced by stimulation of temperature receptors relieves pain caused by ischaemia and the bioelectric effect stimulates the local sensory threshold of pain to return to a normal level (Racz and Ruiz‐Lopez 2006). The degree of penetration into the tissue varies depending on the wave frequency, usually range from 915 to 2450 MHz (Wessapan, Srisawatdhisukul, and Rattanadecho 2011).

Capacitive resistive monopolar radiofrequency (CRMRF) is a low frequency radiofrequency that operates at the clinical setting average 448 kHz (Carralero‐Martínez et al. 2022; Kumaran and Watson 2015, 2019). A recent review presented evidence supporting both continuous and pulsed shortwave therapy in managing CLBP (Farì et al. 2022). A study Tashiro et al. evaluated the effect of CRMRF combined with ET showed a higher efficacy with respect to functional disability and pain (Tashiro et al. 2020).

This study aims to evaluate the effectiveness of 448 kHz CRMRF combined with PNF training in reducing pain and disability among participants with CLBP. A further objective is to compare the efficacy of this combined treatment against PNF training alone in improving functional disability and pain relief. The primary hypothesis posits that the combination of CRMRF and PNF training will significantly reduce pain and disability in CLBP patients compared to PNF training alone. Secondary hypotheses suggest that participants receiving the combined treatment will exhibit greater improvements in functional disability scores, as measured by the Oswestry Disability Index and other disability scales, compared to those receiving only PNF training.

## 2 Methods

### 2.1 Sample Size, Allocation Concealment & Blinding

Power analysis was conducted before recruitment phase using G*Power tool, version 3.1.9.6 (Faul et al. 2007) based on previous research (Farì et al. 2022; Kasimis et al. 2023; Tashiro et al. 2020; Wachi et al. 2022) and expert opinion. Assuming medium effect size (Cohen *f* = 0.25), medium correlation between repeated measures (*ρ* = 0.5), statistical power of 95% and a probability at Type I error at 5%, total sample size needed was 54. To account for the possible dropout, we recruited 15% more patients giving us a total randomised sample of 62 participants. Participants were blinded to allocation, but they were not blinded to difference in therapeutic intervention. Therapists were not blind to the participants allocation to either experimental or control group. Assessors were blind to participant allocated groups, both in baseline and after therapy measurements.

### 2.2 Study Design

The purpose of this single‐blind, parallel (1:1 allocation ratio) randomised controlled trial is to test the effects of CRMRF with PNF exercises versus only PNF exercises alone.

### 2.3 Subjects & Enrolment

The trial was conducted in a privately owned polyclinic with patients either referred form general practitioner or self‐referred. Inclusion criteria were patients with older than 18 years afflicted with CLBP, which is defined as low back pain persisting for 12 weeks or more (Maharty, Hines, and Brown 2024). Exclusion criteria were spinal fractures, radiculopathies, history of spinal surgery, spinal stenosis, history of thrombosis, pacemaker and pregnancy. Ethics approval was obtained by Ethics Committee after planning phase and before recruitment phase. Before inclusion into study every patient gave written consent to participate in study. We adhered to all Ethical Principles for Medical Research Involving Human Subjects by World Medical Association Declaration of Helsinki (World Medical Association 2013). After recruitment, participants were randomised by 1:1 allocation using block randomisation. Randomisation was done off‐site by a statistician and allocation was concealed by opaque envelopes that were opened successively after recruitment of every participant. Last five participants were randomised together after recruitment to prevent recruiter guessing the allocation of last patient.

### 2.4 Interventions

Participants in the experimental group were treated with a combination of 448 kHz CRMRF and PNF, while the control group was treated only with PNF.

CRMRF treatment is a type of medical procedure that uses radiofrequency energy to heat the targeted tissue. Participants in the experimental group were treated by (1) 448 kHz capacitive resistive monopolar radiofrequency for 15 min using INDIBA CT8 device. After CRM treatment, patients exercised using and (2) PNF concept for 20 min. The PNF treatment was administered by rotating the entire length of the spine rather than focusing on individual spinal segments. The upper trunk was rotated to move the right shoulder towards the left ilium, while the left rotation of the lower trunk segment brought the right ilium towards the left shoulder. According to the ‘Chopping’ pattern, this involved bilateral flexion of the legs to the left, and the ‘Lifting’ pattern involved bilateral extension of the legs to the right, directing movements towards extension and flexion (Beckers and Buck 2021; Smedes et al. 2016). Initially, the extremities' movement was blocked until the contraction of the trunk muscles was detected. Once contraction was confirmed, patients were allowed to perform dynamic movements against resistance. This method ensured that while the extremities moved, the trunk muscles contracted to stabilise the trunk. Blocking the terminal movement of the extremities was used to reinforce resistance through leverage directed towards the trunk. Emphasis was placed on trunk muscle contraction during each movement, aiming for elongation rather than lumbar spine extension (Conroy et al. 2023). Participants in the control group were treated only with the PNF concept for 20 min. This control group treatment was identical to the second part of the treatment received by the experimental group. Both treatment groups underwent 10 therapy sessions over two weeks, with each session conducted once per weekday and no with sessions on weekends.

### 2.5 Outcomes

The primary outcomes were pain and functional disability. Pain was measured using standard tool for pain assessment–Visual Analogue Scale (VAS), measuring from 0 to 10 cm (Delgado et al. 2018). Functional disability was measured by Oswestry Low Back Pain Disability Questionnaire (Fairbank and Pynsent 2000). For secondary outcome of disability associated with pain Roland‐Morris Disability Questionnaire (RMDQ) (Roland and Morris 1983) and Quebec Back Pain Disability Scale (QPDS) (Fritz and Irrgang 2001) were used. This secondary outcome focuses on the extent to which pain directly limits the ability to perform movements or activities. The decision to include the secondary outcome of disability associated with pain, in addition to the primary outcome of functional disability, was driven by the intention to assess the multifaceted nature of disability resulting from CLBP. This approach acknowledges that CLBP can hinder functionality not only through pain but also through other factors such as decreased muscle strength and fear of movement, which are not directly related to pain. Validity and reliability of all three instruments is well researched (Davidson and Keating 2002), with ODI having test‐retest reliability of 0.83–0.99, ICC of 0.94 and Cronbach's *α* from 0.74 to 0.87 (Fairbank et al. 1980; Fairbank and Pynsent 2000; Vianin 2008). RMDQ has similarly good metric characteristics, having test‐retest reliability of 0.42–0.91, ICC of 0.74 and Cronbach's *α* of 0.92 (Kersten et al. 2021; Macedo et al. 2011; Ostelo et al. 2004). Finally, QPDS exhibited comparable characteristics of test‐retest reliability of 0.92, ICC of 0.94 and Cronbach's *α* from higher than 0.9 (Kopec et al. 1995; Smeets et al. 2011). The minimal clinically important difference (MCID) varies based on the anchor chosen for anchor‐based calculations of MCID or whether distribution‐based methods were used. For ODI, MCID varies from 8 to 17 with 10 being cited as a minimum by a consensus, for RMDQ, MCID varies from 4 to 5, and for QPDS, MCID varies from 8.5 to 24.6 (Maughan and Lewis 2010; Ostelo et al. 2008; van der Roer et al. 2006).

Basic anthropometric and sociodemographic data was collected, namely height, weight, BMI, marital status, and education.

Finally, all participants were screened by STarT Back Screening Tool (Medeiros et al. 2021; Murphy et al. 2013) after randomisation to assess what are the risks of our randomised samples developing serious disability due to CLBP. Using the STarT Back Screening Tool ensures participant groups are comparable in physical and psychosocial risk factors, enhancing the trial's validity.

Participants were assessed before starting their allocated therapy plan and after completing their respective interventions, one day after last therapy sessions. No additional follow‐up was performed.

### 2.6 Statistical Analysis

After completing all measurements, we conducted an intention‐to‐treat analysis (ITT). Data were analysed using a 2 × 2 mixed‐model ANOVA, where participants were treated as a random variable to account for within‐subject variability, and both group allocation and time were treated as fixed effects. Before applying inferential analysis, models were checked for the assumption of normality of residuals, homoscedasticity, and lack of highly influential outliers. No post‐hoc tests were performed as they are redundant in 2 × 2 designs. Baseline socio‐demographic and clinical characteristics were tested using chi‐square test, Fisher's exact test, and Student's *t*‐test as appropriate. The probability of Type I error was set to 5%. All statistical analyses were conducted using statistical software R (ver. 4.2.2).

## 3 Results/Findings

**FIGURE 1:**
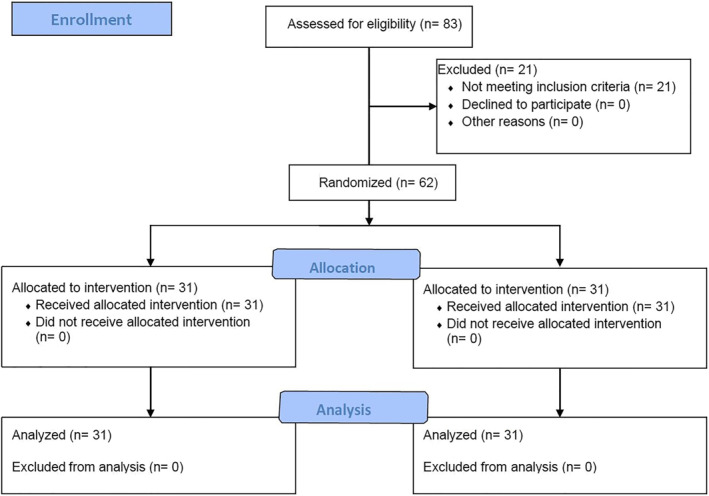
CONSORT flow‐diagram.

The recruitment process lasted for 4 months, between March 2022 and July 2022. A total of 83 participants were screened for eligibility, of which 21 participants were excluded and 62 fulfiled the inclusion criteria and agreed to take part in this study (Figure 1). Thirty‐one participants were allocated either into experimental or control group where they received the intended treatment and were analysed for both primary and secondary outcomes. There were no dropouts from the randomisation, through the intervention, to analysis of the outcomes. No harms our unintended side effects were reported by any participant in either treatment groups.

### 3.1 Descriptive Analysis

Table 1 shows socio‐demographic characteristics of sample. We have tested socio‐demographic characteristics for differences between groups. The only characteristic that showed statistically significant differences between groups was Education variable. Experimental group had larger proportion of people who finished high school and control group had a larger proportion of people who had bachelor's degree. It is likely that this is a spurious difference, with no basis in anything other than simple chance. The STarT Back Tool showed no significant differences in risk of developing CLBP between groups. And although there are more individuals at medium risk in the control group than in the experimental group, we cannot reject the hypothesis that these differences are merely due to chance.

**TABLE 1:** Socio‐demographic characteristics of the samples.

	Experimental group (*n* = 31)	Control group (*n* = 31)	*p*
Gender			
Male	16 (51.6%)	11 (35.5%)	0.31^a^
Female	15 (48.4%)	20 (64.5%)	
Age			
Mean (SD)	48.9 (17.1)	50.7 (17.6)	0.69^b^
Height (cm)			
Mean (SD)	170 (11.3)	169 (11.2)	0.80^b^
Weight (kg)			
Mean (SD)	76.9 (13.2)	78.1 (16.1)	0.74^b^
BMI			
Mean (SD)	26.6 (4.12)	27.1 (4.24)	0.60^b^
STarT back tool			
Low risk	23 (74.2%)	17 (54.8%)	
Medium risk	5 (16.1%)	12 (38.7%)	0.15^c^
High risk	3 (9.7%)	2 (6.5%)	
Marital status			
Married	19 (61.3%)	20 (64.5%)	0.99^c^
Divorced	1 (3.2%)	1 (3.2%)	
Widow(er)	4 (12.9%)	4 (12.9%)	
Single	7 (22.6%)	6 (19.4%)	
Education			
Elementary	1 (3.2%)	0 (0%)	0.03*^c^
High school	19 (61.3%)	12 (38.7%)	
Bachelor's	5 (16.1%)	15 (48.4%)	
Master's	6 (19.4%)	4 (12.9%)	

*Note: p* = *p* value.

^a^ Chi‐square test.

^b^ Student's *t*‐test.

^c^ Fisher's exact test.

*Statistical significance (*p* < 0.05).

Table 2 shows baseline and after intervention scores for pain and disability. To increase certainty that treatment groups are balanced in terms of the primary and secondary outcomes we analysed clinical baseline outcomes between groups and there were no statistically significant differences (Table S1).

**TABLE 2:** Clinical outcomes for two intervention groups.

Time point	Experimental group (*n* = 31)		Control group (*n* = 31)	
	Baseline	After	Baseline	After
VAS (cm)	6.81 (1.45)	2.00 (1.55)	6.35 (1.40)	4.00 (1.93)
ODI	38.6 (14.3)	10.4 (12.0)	37.2 (13.5)	26.1 (14.8)
QPDS	54.5 (16.4)	19.3 (17.0)	55.5 (14.7)	40.1 (20.9)
RMDQ	11.2 (5.14)	2.94 (2.94)	10.8 (4.42)	8.00 (3.66)

*Note:* All values are mean (SD).

### 3.2 Inferential Analysis

**FIGURE 2:**
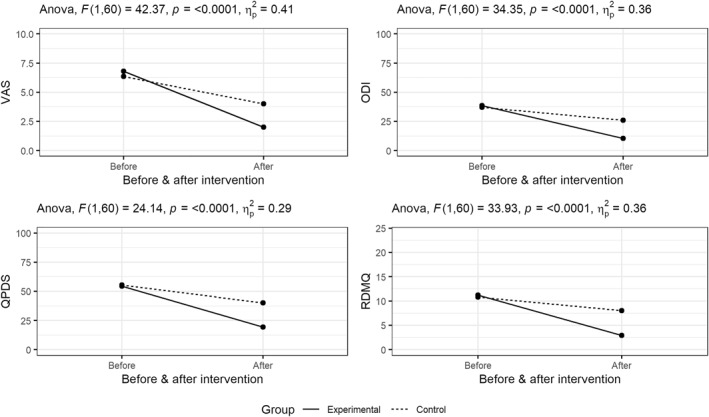
Interaction plots for all outcomes. $\eta_{p}^{2}$ = partial eta squared (effect size); *F* = *F* ratio; *p* = *p* value.

Table 3 shows the results of mixed‐model ANOVA. All clinical outcomes had statistically significant interaction effects at *p*‐value < 0.001. The effect size, partial eta‐squared ($\eta_{p}^{2}$), is 0.41, 0.36, 0.29 and 0.36 for VAS, ODI, QPDS and RMDQ, respectively. In accordance with Cohen's rule of thumb (Cohen 1973) all effect sizes are large. Two primary outcomes–VAS and ODI show a much larger decrease in value in the experimental group. For the experimental group VAS scores have on average decreased by 2.46 more than the participants in the control group. For ODI, scores in the experimental group decreased by 17.1 points more than in the control group. QPDS and RMDQ had an average larger decrease in the experimental group than in control group by 19.8 and 5.46 points, respectively. Effects of the treatment groups across time for all outcomes is visualised on an interaction plot (Figure 2).

**TABLE 3:** ANOVA (mixed‐model, df = 1.60).

Variable	Group			Time			Interaction		
	*F*	*p*	$\eta_{p}^{2}$	*F*	*p*	$\eta_{p}^{2}$	*F*	*p*	$\eta_{p}^{2}$
VAS	4.65	0.04*	0.07	361.50	< 0.001*	0.86	42.37	< 0.001*	0.41
ODI	5.10	0.03*	0.08	181.42	< 0.001*	0.75	34.35	< 0.001*	0.36
QPDS	7.62	0.09	0.13	155.70	< 0.001*	0.72	24.14	< 0.001*	0.29
RMDQ	6.23	0.02*	0.09	141.41	< 0.001*	0.70	33.93	< 0.001*	0.36

Abbreviations: $\eta_{p}^{2}$ = partial eta squared (effect size), *F* = *F* ratio, *p* = *p* value.

*Statistical significance (*p* < 0.05).

## 4 Discussion

The management of CLBP presents a multifaceted challenge, necessitating a multidisciplinary approach to treatment that effectively addresses both pain and disability. Our research has provided valuable insights into the efficacy of combining CRMRF at 448 kHz with PNF exercises, shedding light on a promising therapeutic strategy for CLBP. This discussion aims to contextualise our findings within the broader landscape of CLBP management, particularly focusing on the significance of our results in relation to the minimally clinically important difference (MCID) for various disability instruments.

Our study demonstrated that patients receiving CRMRF combined with PNF exercises experienced superior outcomes in both pain reduction and functional improvement compared to those who did not receive CRMRF. The decrease in pain, as measured by the Visual Analogue Scale (VAS), exceeded the MCID of 2.0 cm (Ostelo and de Vet 2005), indicating not only statistical significance but also clinical relevance. This aligns with previous research (Tashiro et al. 2020; Wachi et al. 2022) who also reported significant improvements in VAS scores among CLBP patients treated with CRMRF and exercise therapy.

Further, our investigation extended to assess the impact of the treatment on disability, utilising the Oswestry Disability Index (ODI), Quebec Pain Disability Scale (QPDS), and Roland‐Morris Disability Questionnaire (RMDQ). The improvements observed in these instruments were not merely statistically significant; they also surpassed the MCID, highlighting the clinical importance of our findings. The ODI difference of improvement in favour of the experimental group was significantly above the MCID of 10 points for individuals with CLBP. For the QPDS, the difference in change scores was significantly also exceeded the MCID of six points. Finally, the difference in change RMDQ changes scores between groups was also larger than the MCID of 3.5 points. This substantial improvement suggests that the combination of CRMRF and PNF exercises can lead to meaningful improvements in daily living activities and overall function which has some support in the previous similar studies on CRMF combined with exercise (Tashiro et al. 2020) or manual therapy (Kasimis et al. 2023).

These findings are not isolated nor limited to CLBP, they resonate with the broader body of evidence supporting the efficacy of CRMRF in conjunction with various therapeutic exercises for CLBP and other musculoskeletal disorders. For instance, several studies (Albornoz‐Cabello et al. 2020; Carralero‐Martínez et al. 2022; Dinçer et al. 2024; Kumaran and Watson 2019; Taheri, Sadri, and Maghroori 2023) have all highlighted the benefits of CRMRF in reducing pain and improving function across different patient populations.

The integration of CRMRF with PNF exercises offers a novel approach that may enhance the effectiveness of each modality. CLBP is often characterised by a combination of factors, including inflammation, muscle tension, tissue damage, and impaired blood flow, which contribute to persistent pain and functional disability. CRMRF works by generating deep tissue heating, which enhances blood circulation and promotes tissue healing. The increased blood flow helps to deliver essential nutrients and oxygen to the affected areas, facilitating the repair of damaged tissues and reducing muscle stiffness. Moreover, the deep heating effect of CRMRF helps to relax tight muscles, reducing muscle tension and alleviating pain. Furthermore, CRMRF can enhance cellular metabolism and regeneration and this effect helps to strengthen the lumbar muscles and ligaments, providing better support to the spine and reducing the likelihood of recurrent pain episodes (Kumaran, Herbland, and Watson 2017). The potential mechanisms underlying this synergy could include the deep heating effects of CRMRF, which may improve tissue extensibility, reduce viscosity, and promote blood flow, thereby augmenting the effects of PNF exercises. However, further research is needed to fully understand these mechanisms and to optimise treatment protocols.

In conclusion, our study contributes to the growing evidence supporting the use of CRMRF in combination with PNF exercises for the treatment of CLBP. The observed improvements in pain and disability, as measured by VAS, ODI, QPDS and RMDQ, not only achieve statistical significance but also surpass the MCID, underscoring their clinical relevance. These findings suggest that this therapeutic combination could offer a valuable non‐invasive treatment option for individuals suffering from CLBP, with the potential to significantly enhance their quality of life. Future research should focus on exploring long‐term outcomes, expanding the application to other musculoskeletal conditions, and explaining the therapeutic mechanisms at play.

### 4.1 Limitations

Despite the promising outcomes reported in our study, it is crucial to acknowledge certain limitations that could impact the interpretation and generalisability of our findings. Firstly, the absence of a placebo group presents a significant limitation. Due to the inherent characteristics of CRMRF treatment, specifically its distinctive heating effect, it was not feasible to implement a convincing placebo condition that could replicate the sensation without delivering the actual therapeutic effect. This limitation is notable because the absence of a placebo control makes it challenging to fully discount the placebo effect, which could potentially contribute to the observed benefits. The psychological impact of receiving a novel or technologically advanced treatment can influence patient‐reported outcomes, thus the specific effects attributable solely to CRMRF, independent of placebo responses, remain uncertain.

Secondly, the lack of mid‐term and long‐term follow‐up assessments in our study restricts our understanding of the durability of treatment effects. Without these follow‐up data, it is difficult to ascertain whether the improvements in pain and functional capacity observed post‐treatment are sustained over time. This is a critical consideration for chronic conditions like CLBP, where the long‐term management of symptoms is a key concern for both patients and clinicians. Future research efforts should aim to include placebo or sham‐controlled conditions, when feasible, and incorporate follow‐up evaluations to capture the longevity of treatment benefits, thereby providing a more comprehensive understanding of the therapeutic value of CRMRF combined with PNF exercises in the management of chronic low back pain.

Thirdly, given fairly low sample size and single centeredness of this study, limitations are imposed on the ability to generalise the results we present.

## 5 Implications on Physiotherapy Practice

The combined use of CRMRF and PNF has demonstrated significant improvements in pain reduction and functional disability, exceeding the MCID for key outcome measures such as the VAS, ODI, QPDS, and RMDQ. This suggests that CRMRF, when used as an supplemental modality to PNF, can provide substantial clinical benefits, making it a valuable addition to the physiotherapist's toolkit for managing CLBP. By incorporating CRMRF, physiotherapists can potentially speed‐up recovery and improve the overall efficacy of the PNF exercise protocol.

Additionally, the study's results highlight the importance of adopting a multimodal approach to CLBP treatment. Physiotherapists should consider integrating CRMRF with other exercise therapies, not limited to PNF, as it is likely that CRMRF would positively impact other exercise‐based rehabilitation programs. In practice, physiotherapists can implement CRMRF as a preparatory treatment before conducting PNF exercises or other therapeutic modalities. This sequence can maximise the benefits of physical rehabilitation by ensuring that the tissues are optimally prepared for stretching, strengthening, and proprioceptive training.

## Ethics Statement

Ethics approval was obtained by Ethics Committee of University of Applied Health Sciences, Zagreb, Croatia (KL: 602‐04/21‐18/544, URBR: 251‐379‐10‐21‐02).

## Consent

Informed consent was obtained from all the participants before their enrolment in the study.

## Conflicts of Interest

The authors declare no conflicts of interest.

## Permission to Reproduce Material From Other Sources

The authors have nothing to report.

## Supporting information

Table S1

## Data Availability

Data is available upon reasonable request from the corresponding or the first author.
