# Supplementary material for: Evaluating the Efficacy of Capacitive Resistive Monopolar Radiofrequency Combined With Proprioceptive Neuromuscular Facilitation in Managing Chronic Low Back Pain: A Randomised Controlled Trial
Source: Physiother Res Int. 2024 Nov 21;30(1):e70009. doi: 10.1002/pri.70009 (PMC11582020; doi:10.1002/pri.70009)
Supplement: Supplementary file 1 — Table S1 [file PRI-30-e70009-s001.docx]

**Table S1.** Statical testing of baseline clinical outcomes

|  | **Experimental (n=31)** | **Control (n=31)** | **t-statistic** | **p-value** |
| --- | --- | --- | --- | --- |
| VAS (cm) | 6.81 (1.45) | 6.35 (1.40) | 1.25 | 0.22 |
| ODI | 38.6 (14.3) | 37.2 (13.5) | 0.40 | 0.69 |
| QPDS | 54.5 (16.4) | 55.5 (14.7) | -0.23 | 0.82 |
| RMDQ | 11.2 (5.14) | 10.8 (4.42) | 0.32 | 0.75 |

**Note:** All values are mean (SD)
